# Using international human rights law to improve child health in low-income countries: a framework for healthcare professionals

**DOI:** 10.1186/s12914-016-0083-1

**Published:** 2016-03-30

**Authors:** Bernadette Ann-Marie O’Hare, Delan Devakumar, Stephen Allen

**Affiliations:** School of Public Health and Paediatrics and Child Health, College of Medicine, University of Malawi, Blantyre, Malawi; Global Health Implementation, University of St Andrews, St Andrews, Scotland; Institute of Epidemiology and Health Care, UCL, London, UK; Department of Medical Sciences, Liverpool School of Tropical Medicine, Liverpool, UK

**Keywords:** Human rights, Children, Health professionals, Health, Duty bearers, United Nations Convention on the Rights of the Child

## Abstract

**Background:**

The Committee on Economic, Social and Cultural Rights states that the right to health is closely related to, and dependent upon, the realization of other human rights, including the right to food, water, education and shelter which are important determinants of health. Children’s healthcare workers in low income settings may spend the majority of their professional lives trying to mitigate deficiencies of these rights but have little influence over them. In order to advocate successfully at a local level, we should be aware of the proportion of children living in our catchment population who do not have access to their basic rights. In order to carry out a rights audit, a framework within which healthcare workers could play their part is required, as is an agreed minimum core of rights, a timeframe and a set of indicators.

**Discussion:**

A framework to assess how well states and their developmental partners are adhering to human rights principles is discussed, including the role that a healthcare worker might optimally play. A minimum core of economic and social rights seeks to establish a legal minimum set of protections, which should be available with immediate effect and applicable to all nations despite very different resources. Minimum core rights and the impact that progressive realisation may have had on the right to health is discussed, including what they should include from the perspective of children’s health. A set of absolute rights are suggested, based on physiological needs and aligned with the corresponding articles of the United Nations Convention on the Rights of the Child. The development indicators which are likely to be used to monitor progress towards the Sustainable Development Goals is suggested as a way to monitor rights. We consider the ways in which the healthcare worker could use a rights audit to advocate with, and for their community.

**Summary:**

These audits could achieve several objectives. They may legitimise healthcare workers’ interests in the determinants of health and, as they are often highly respected by their community, this may facilitate them to be agents for change at a local level. This may raise awareness on basic human rights and their importance to health and contribute to a needed change in mind-set from one of development needs to absolute rights. The results may catalyse colleagues to analyse further the upstream reasons why children, and the families in which they live, are not having their rights met.

## Background

Human rights have been enshrined in the Universal Declaration of Human Rights (UDHR) adopted in 1948 [1] and codified in a series of international treaties and instruments which have been ratified by most countries [2]. The rights and obligations of the UDHR are further characterised by two subsequent covenants; the International Covenant on Civil and Political Rights, ratified by 168 countries and the International Covenant on Economic on Social and Cultural Rights (ICESCR), ratified by 164 countries. General Comment Number 14 of the Committee on Economic, Social and Cultural Rights states that the right to health is not confined to the right to healthcare and dependent upon the realization of other rights including the right to food, water, education and shelter and states must ensure equal access for all to the underlying determinants of health, including immunization programmes [3]. The economic and social rights for children are also asserted in article 4 of the United Nations Convention on the Rights of the Child (UNCRC) [4], ratified by 195 countries.

As healthcare workers in low income settings, we accept that factors over which we have little influence, such as income, food supply, drinking water, sanitation and maternal education have a significant impact on children’s health. As a result, we are in the invidious position of having little influence over the determinants of health, but spend our professional lives dealing with the consequences of deficiencies in these basic needs. In order to advocate successfully at a local level, we should be aware of the proportion of children living in our catchment population who do not have access to their basic rights. We suggest that the role which a healthcare worker might optimally play is to audit the outcome indicators of the rights of children living in their catchment area. This audit ideally should be done within a larger framework, which also analyses the upstream influences on these rights. In order for healthcare workers to audit access to basic rights, there needs to be an agreed set of minimum core obligations, a timeframe when they should be available and a set of indicators to measure outcomes. We discuss each of these issues in turn and review possible ways to use the results of the audit to advocate at a local level.

## Discussion

### Frameworks to assess human rights

There are several frameworks to assess how well states are adhering to human rights principles. The main components include; outcome indicators which evaluate key variables such as child mortality, indicators which evaluate a state’s conduct, including if a state has ratified and legislated for a given right and the effort towards implementation such as national guidelines and resource allocation [2]. The Centre for Economic and Social Rights use a framework whereby they look at Outcomes, Policy Efforts, Resources and finally do an Assessment (OPERA), Table 1 [5]. Assessing policy effort, resource generation and allocation is probably beyond what most healthcare workers would have time and possibly expertise to do. Attributing responsibility for high rates of child mortality involves complex analyses of conduct and resources [5] and is especially difficult as the raising and allocation of resources in poor countries may not be entirely self-determined and the role of other organisations such as international financial organisations will need to be considered [6]. Nonetheless auditing of outcomes, the top row in the OPERA framework, and advocacy at a local level could play a crucial role and possibly catalyse further analysis of other components of the pathway by groups such as public health professionals or civil society.

**Table 1:** The OPERA framework for assessing compliance with human rights [5]

Outcome indicators	Measure aggregate levels of rights enjoyment (Minimum core obligations)	Measure disparities in rights enjoyment (non-discrimination)	Measure progress over time (Progressive realisation)
**Policy** **E** **fforts**	Identify legal and policy commitments	Examine policy content and implementation	Analyse policy progress
**R** **esource indicators**	Evaluate resource allocation	Evaluate resource generation	Analyse policy processes
**A** **ssessment**	Identify other determinants	Understand state constraints	Determine state compliance

Bold data are the headings

### A minimum core

A minimum core of Economic, Social and Cultural rights (ESCR) seeks to establish a legal minimum set of protections, which should be available with immediate effect and applicable to all, in all nations, despite different resources. International human right institutions have tried to compensate for the loophole of progressive realisation by establishing a minimum core of rights which are of immediate effect [7]. The attraction of the concept is that if an international minimum core could be agreed there would be a higher burden of proof on states to prove that they are using all available resources to achieve this [5]. Gordon and colleagues assert that a rights based strategy will only reduce child mortality if some rights are prioritized over others [8] and others have argued that a stronger minimum core could make significant contribution towards global health equity [7]. The Committee on Economic, Social and Cultural Rights (CESCR) has suggested that an international minimum core of ESCR should be developed and be legally binding, but reaching a consensus on minimal core rights has followed a meandering path [9].

From the perspective of healthcare workers, a set of minimum core obligations could be powerful, as these are critical determinants of health, many of which are also basic human rights [10]. Seventy years ago Maslow recognised that needs are hierarchal and the most basic needs are our physiological needs required for survival, i.e., air, water, food and shelter. The other groups of needs are for safety, love, esteem and self-actualization [11]. These are often represented as tiers on a pyramid of needs, with the physiological needs on the lowest tier. If physiological needs are not met, a child will not survive, much less thrive to enjoy their other rights.

Therefore we concentrate on the basic or physiological needs which are also human rights. We have called them ‘absolute rights’, the right to food, water, sanitation and shelter. Female education is very important to children having the right to life; every extra year of education of females of reproductive age results in a 9.5 % decrease in child mortality [12]. Therefore we have included Article 28 of the UNCRC, the right to free primary school education as an absolute right. The right to healthcare is complicated by where to place the bar in terms what services should be provided and who should pay for them. However it is known that basic vaccinations reduce mortality in the first year of life [13], and as in General Comment number 14 [3], we have included basic vaccinations as an absolute right for all children.

### Progressive Realisation

When should a minimum core of rights come into effect? The Office of the United Nations High Commissioner for Human Rights (OHCHR) has stated that Civil and political rights should be immediately applicable but Economic, Social and Cultural Rights are subject to progressive realisation, If resources are lacking [2]. However, it is likely that progressive realisation has undermined the right to health and diminished both domestic and international responsibilities [7]. General Comment Number 3 of the CESCR (1990) has stated that obligations of results may be achieved progressively while the obligation of conduct is immediate [5, 14]. General Comment Number 14 (2000) states that core obligations on the right to health are of immediate effect [3, 8]. In our quest for a minimum core of rights, which are independent of a state’s resources and less likely to be undermined by the principle of progressive realisation, we believe the absolute right to food, water, sanitation, shelter, basic education and vaccinations are indisputable and that these should be of immediate effect. In circumstances of limited resources (as opposed to unequal distribution of resources), these absolute rights should be within the Extra Territorial Obligations of the international community [15].

### What indicators?

“A major concern with the use of indicators for human rights assessments stems from the fact that there is not a significant body of work in the literature, or in practice, that uses a consistent and coherent frame-work to identify and develop those indicators” [2], in addition indicators are needed that assess the right to health [16]. While there is a difference between a human right and a development goal, there is no clear distinction between quantitative ESCR outcome indicators and development indicators in terms of their collection [17]. Data collected by development agencies can be used as indicators to benchmark the outcome component, or the results, of adherence to human rights obligations [2]. The OHCHR has noted that ESCR are resource intensive, subject to progressive realisation and should be reported and perceived as positive human rights [18]. International development agencies often collect development goals as positive, (i.e., the percentage of people who do have access to …). When development data, such as the percentage of people *who have access to* improved water, is used for the purposes of monitoring an absolute right, it should be reported appropriately i.e., the percentage of people *who do not have access* to …. This brings a different response; who are the duty bearers and what particular obligation has the duty bearer failed to respect, protect or fulfil? This may help shift the dialogue and more importantly the mind-set, from one of charity to one about rights.

For healthcare workers to audit access to human rights, indicators for each right which is considered absolute is required. We have mapped, what we consider to be absolute and immediate rights, as they are set out in the UNCRC, to the global monitoring indicators suggested by the Open Working Group (OWG) for the sustainable development goals [19] and the UNHCR [2], using the outcome domain of the OPERA framework, Table 2.

**Table 2:** Absolute rights in each of the domains and indicators from the SDG

	States shall [4]	SDG suggested indicator [19]	SDG description of indicator [19]	Absolute rights
**Water**	provide access to clean drinking water Article 24 (2c)	Percentage of population using safely managed water services, by urban/rural	Basic drinking water sources can include: piped drinking water supply on premises; public taps/stand posts; tube well/borehole; protected dug well; protected spring; rainwater; and bottled water with a total collection time of 30 min or less for a round trip including queuing.	Percentage of children (in the sample) who do not have access to drinking water from any of the listed sources or whose collection time is >30 min.
**Sanitation**	ensure that all sections of society are informed about and have access to sanitation Article 24 (2e)	Percentage of population using safely managed sanitation service by urban/rural	The following facilities are considered adequate if the facility is not shared with other households: a pit latrine with a superstructure, and a platform or squatting slab constructed of durable material (composting latrines, pour-flush latrines, etc.); a toilet connected to a septic tank; or a toilet connected to a sewer network (small bore or conventional).	Percentage of children (in the sample) who share a latrine with other households or who do not have access to any of the listed options
**Food**	take measures within their means to assist parents or carers to provide adequate nutrition Article 27 (3)	Prevalence of stunting and wasting in children under 5 years of age	Prevalence of stunting and wasting in children < 5 years, using WHO standards [21]	Percentage of children (in the sample) who are stunted as described or wasted as described or both
			Stunting is low height for age; children < 5 years whose height for age is two or more standard deviations below the median.	
			Wasting is low weight for age; children < 5 years whose weight for age is two or more standard deviations below the median.	
**Shelter**	take measures within their means to assist parents or carers to provide adequate shelter Article 27 (3)	Safe and affordable housing	Security of tenure (evidence of documentation to prove secure tenure status or de facto or perceived protection from evictions)	Percentage of children (in the sample) who do not live in houses with secure tenure, or which are not durable and with more than two people per room (divide number of occupants by number of rooms)
			Durability of housing (permanent and adequate structure in non-hazardous location)	
			Sufficient living area (not more than two people sharing the same room)	
**Education**	make primary education compulsory and available free to all; Article 28(a)	Primary completion rates for girls and boys	Primary completion rates for girls and boys	Percentage of boys and girls who have dropped out of school without completing primary school education.
**Health**	to develop preventive health care Article Article 24(F)		Percent of children receiving full immunization (as recommended by national vaccination schedules)	Percent of infants aged 1 year who have not received full immunization (as recommended by national vaccination schedules)

Bold data are the headings

### The role of the health professional

Health workers can play a powerful role by reporting the absolute rights of children living in their catchment area using the outcome domain of the OPERA framework, top row, Table 1. For a district or a country, routinely collected data such as demographic and health surveys [20], can also be used to report absolute rights and may also include data such as neonatal, infant and child mortality as, where information is collected carefully and the data sets are sufficiently large this is meaningful. It is important to disaggregate the data to compare between groups such as children from different districts, rural and urban, and male and female. Data on each of these indicators is relatively easy to collect. For example, at one of the author’s institutions, this information is collected for all unscheduled attendees, using an electronic patient record, see Table 3. Vaccination and nutritional status data is also collected for a subset of patients.

**Table 3:** Example of a questionnaire to assess absolute rights (Bold indicates that absolute right has not been met)

Name	
Address (or GPS coordinates)	
DOB or year of birth	M/F (circle)
WATER	(circle)
Where do you get your water?	piped drinking water supply on premises
	public tap/stand post
	tube well/borehole
	protected dug well
	protected spring; rainwater
	bottled water
	**Surface water**
How long does it take you to collect your water, including queuing?	**>30 min/<** 30 min; (circle)
SANITATION	(circle)
Does your house share the toilet with another household?	No/**Yes**
What sort of toilet do you use (circle)?	a pit latrine with a structure, and a platform or squatting slab
	a toilet connected to a septic tank or a sewer network
	**no toilet/open defaecation**
FOOD	
Child's age in months	
Child's height	
Child's weight	
Height/age or weight/height >2SD below median or MUAC <12.5 cm (6-59 months)?	**Yes**/No (circle)
SHELTER	
Is the house durable in terms of structure?	**No**/Yes (circle)
Is the house durable in terms of tenure?	**No**/Yes (circle)
How many people live in your house?	
How many rooms are there?	
Number of people/room?	Less than 2, 2, **more than 2** (circle)
EDUCATION	
How many years did mother/guardian attend school?	1 2 3 4 5 6 7 8 9 10 11 12 13 14 (circle)
If child has left school, did she/he complete primary school?	**No**/Yes (circle)
HEALTH	
Vaccine schedule complete at 12 months (National Guidelines)?	**No**/Yes (circle)

These audits could achieve several objectives. They may raise awareness of basic human rights and their importance to health. Presenting the results could give the healthcare worker an informed voice to advocate at forums such as district councils and legitimise their role as agents for change. The use of the results will vary according to local circumstances. Advocacy and budgetary oversight at a local level, by a group who are often highly respected in their community is important, as resource allocation is critical and this may channel allocation toward human rights for health. Absolute rights may provide local councils and health teams some leverage when negotiating with international organisations and non-governmental organisations (NGO). Donors and NGOs often arrive in a district with a predetermined set of objectives. A set absolute rights and local data, could allow local teams to ask partners to prioritise these; the scale up of access to absolute rights might be significant.

The availability of ESCR rights are highly influenced by national resource generation and allocation; i.e., what happens locally is influenced by national and international policies and markets. Healthcare workers could work with other professionals such as public health and civil society to do a complete OPERA assessment. Local level data could influence and be used to advocate for changes in national level policy. Governments that ratify the human rights treaties have a legal obligation to advance the rights within them. This includes the regulation of their local and regional sub-divisions and other non-governmental organisations and actors, as well as fiscal and monetary policies. If unable to meet rights obligations, then the government is obliged to seek support and high-income countries have a legal obligation to provide cooperation. A set of absolute rights would place a higher burden of proof on outside state parties to prove that they were adhering to their extra territorial obligations by cooperating with countries to achieve a minimum core of rights for all. This could include cross border tax arrangements, trade agreements including intellectual property and services, and the movement of capital. A set of absolute rights could contribute to a needed change in mind-set from one of development needs to absolute rights. Concerns that an absolute rights approach may result in resources being diverted from other important human rights should be allayed by the principle of non-retrogression; states cannot regress in terms of achievements, which is inherent in all human rights principles.

## Summary

Health professionals spend much of their professional lives dealing with the consequences of human rights abuses with little influence over them. We have discussed a framework within which healthcare workers can carry out an audit and be agents for change at a local level. We discuss the minimum core ESCR and the role that progressive realisation may have played in undermining the right to the determinants of health. We suggest absolute rights from the perspective of physiological needs and indicators to monitor them. The proposed audit takes the issues outside the clinic or ward, upstream to the socioeconomic determinants of health and to the decision makers at district level. In many countries health professionals form the largest and best educated group in civil society and are generally respected members of the community. They are uniquely placed to highlight important health problems and to instigate and lead these initiatives in the interest of improving child health. As children live in families, this will also improve population health.

### PANEL

#### Scenario - Community healthcare staff

You are a healthcare worker in a government clinic. The majority of your patients come from poor homes, their access to clean water is erratic and supply of nutritious food is inconsistent. The children you see return to the environment from whence they came so, at best, you are providing a “sticking plaster” and feel frustrated that you are not addressing their fundamental problems. How can healthcare workers influence these factors which you feel are contributing hugely to the morbidity and mortality which you encounter? It is at this stage that many of us give up, overwhelmed by clinical work and unsure of our remit outside of the clinic. However, we could select a sample from the community which our clinic serves (community health workers are ideally placed to do this) and carry out an audit using an absolute rights questionnaire. An alternative approach, although likely to give a less representative sample of the community, would be to audit children who present to the clinic (sick children are less likely to have their absolute rights met). An example of such a questionnaire is shown in Table 3. As these are absolute rights rather than development goals, indicators should be expressed negatively i.e., the percentage of children who do not have access to each absolute right. Collecting identifying data on participants is not needed for this kind of audit, and it may be wisest to avoid it in order to maximise participant confidentiality and ethical practice. Be guided by your local institutional guidelines and managers.

#### Conduct indicators of duty bearers – Policy, effort and resources

Having ascertained the proportions of children living in your catchment area who are not having individual absolute rights met, the next step is to ask “*why are these particular children disadvantaged and who are the duty bearers?” and* then try to advocate for them by going through local structures. For example, in Malawi concerns about water and sanitation could be discussed with the Environmental Health Officer who plays a critical role in the District Health Management Team (DHMT) and concerns about nutrition or education could be raised at regular district development committees. It may be that the findings from the audit catalyses a response from community action groups who may be able to implement a solution or advocate for a change in local policy. Responding to the findings may involve working with the local coordinator for nutrition, water and sanitation or the district council. It may also involve attending local meetings to raise your concerns or working with civil society groups to ensure that district budget allocations are transparent and participatory. The results of your audit may allow your DHMT to request an international organisation to prioritise access to absolute rights such as clean water and sanitation, over other projects, which may be important, but less critical to survival.

## Abbreviations

CESCR: Committee on Economic, Social and Cultural Rights
ESCR: Economic, Social and Cultural Rights
ESR: Economic and Social Right’s
ICESCR: International Covenant on Economic, Social and Cultural Rights
OHCHR: Office of the High Commissioner on Human Rights
OPERA: Outcomes, Policy Efforts, Resources and finally do an Assessment
UDHR: Universal Declaration on Human Rights
UNCRC: United Nations Convention on the Rights of the Child
UNHCR: United Nations High Commissioner for Human Rights
